# Relationship trajectories of pregnant women with their parents and postpartum depression: A hospital-based prospective cohort study in Japan

**DOI:** 10.3389/fpsyt.2022.961707

**Published:** 2022-11-03

**Authors:** Shuhei Terada, Satomi Doi, Yukako Tani, Yuto Maeda, Aya Isumi, Junichi Sugawara, Kazuhisa Maeda, Shoji Satoh, Nobuaki Mitsuda, Takeo Fujiwara

**Affiliations:** ^1^Department of Global Health Promotion, Tokyo Medical and Dental University, Bunkyo, Japan; ^2^Japan Society for the Promotion of Science (JSPS), Tokyo, Japan; ^3^Division of Feto-Maternal Medical Science, Department of Community Medical Support, Tohoku Medical Megabank Organization, Tohoku University, Sendai, Japan; ^4^Department of Obstetrics and Gynecology, Shikoku Medical Center for Children and Adults, Zentsuji, Japan; ^5^Maternal and Perinatal Care Center, Oita Prefectural Hospital, Oita, Japan; ^6^Department of Maternal Fetal Medicine, Osaka Women's and Children's Hospital, Izumi, Japan

**Keywords:** parent-daughter relationship, postpartum depression, pregnancy, social support, trajectory

## Abstract

**Backgrounds:**

A history of childhood abuse and subsequent poor relationship with parents in adulthood among pregnant women is a known risk factor for postpartum depression (PPD). Although parent-daughter relationship can change during pregnancy, little is known whether the trajectories have an impact on PPD. The aim of this study is to examine whether trajectories of parent-daughter relationship during pregnancy are associated with PPD in Japanese mothers.

**Methods:**

In a hospital-based prospective cohort study conducted in Japan, 4,772 women were followed from their first visit to their 1-month postpartum check-up (follow-up rate: 77.4%). Parent-daughter relationship was assessed whether participants were satisfied with their parents at first visit and after delivery. We defined four parent-daughter relationship trajectory categories: consistently satisfied, improving, deteriorating, and consistently unsatisfied. PPD was assessed by the Edinburgh Postnatal Depression Scale. Logistic regression model was applied to adjust covariates.

**Results:**

There were 129 (2.7%), 122 (2.6%), and 181 (3.8%) cases of improving, deteriorating, and consistently unsatisfied relationship, respectively. Compared to the group that was consistently satisfied, pregnant women of the deteriorating and consistently unsatisfied group showed 2.81 (95% CI: 1.73–4.55) and 2.39 (95% CI: 1.58–3.62) times, respectively, more likely to show PPD after adjustment for confounders.

**Conclusion:**

Women who felt that their relationship with parents “deteriorated” or was “consistently unsatisfactory” during pregnancy showed significant risk of PPD. Paying attention to the pregnant women's feelings about the relationship with their parents and promoting positive change may help predict and prevent PPD.

## Introduction

Postpartum depression (PPD) is often defined as an episode of moderate to severe depression that occurs in the postpartum period (1). The global prevalence of PPD was 17% among healthy mothers (2), making it one of the most common morbidities during the perinatal period (3). PPD has been described as “a dangerous thief” that robs motherhood because it deprives women of the precious time with the anticipated baby (4). Moreover, it is associated with behavioral, cognitive, and health-related consequences for offspring (1). In extreme cases, PPD can lead to fatal conditions for mother and child through suicide and infanticide (5, 6). Given the negative impact both for mothers and children, prevention of PPD is warranted. Established risk factors for PPD are prenatal depression, stressful life events, lack of social support, and marital dissatisfaction (3).

Among them, childhood abuse history is known to be a strong risk factor for PPD (7–11). In a cross-sectional study in Spain, a history of childhood physical abuse increases the risk of depressive symptomatology by five times in the earlier postpartum (11). In conjunction with childhood abused history, the association between the parent-daughter relationship and health problems has been also reported throughout the life course. In studies of adolescents, for example, poor parent-daughter relationship has been shown to be associated with eating disorder (12), risky sexual behavior (13, 14), alcohol use (15), and suicide attempt (16). In a study of adults, women who continued to perceive lower levels of closeness with parents had higher risk of psychological distress in adulthood (17). Meanwhile, intimate relationship with mothers plays an important role in promoting self-management of chronic illness (18, 19). Thus, good relationship with parents is positively associated with better well-being throughout the life course (20, 21). To prove this theory, several studies have examined the association between parent-daughter relationship and PPD, and revealed that relationship problems with parents was a risk factor for PPD (22–27). However, these studies only examined the relationship with parents at one time point either before or during the pregnancy. Further, because it is considered that parent-daughter relationship cannot be changed and effective preventive approaches for PPD should be based on modifiable factors (28), few studies focused on the satisfaction with the relationship of pregnant and postpartum women with their parents in the context of prevention of PPD.

Recently, some psychologic theories suggested that pregnant women's relationship with their parents can change during the transition to parenthood (29–31). A qualitative study conducted in the US showed that pregnant women are supposed to feel closer to their mothers and have less conflict in their relationship than before the pregnancy (32). On the contrary, a cross-sectional study among Japanese pregnant women suggested that about 20% reported lower closeness with and dependency on their mothers during pregnancy (33). Moreover, parent-daughter relationship can be modifiable and a potential target for intervention: health care providers may be able to help facilitate positive change in the parent-daughter relationship (32). Therefore, it is necessary to examine the association between the trajectories of parent-daughter relationship and PPD as a basis for the future development of preventive measures for postpartum depression.

Available resources of postpartum period differ across countries (34, 35). In Japan, the proportion of fathers taking childcare leave was only 6.2% in 2018 (36). As for public services, about half of the municipalities provide postpartum physical and mental care, but at most 3% of the population used it in 2019 partially due to a lack of publicity and the complex system (37, 38). Given these circumstances, Japanese women often seek help from their own parents at postpartum. In fact, a cross-sectional survey among Japanese postpartum women has shown that they mostly rely on their biological parents for domestic duties and childcare consultation (38). Also, there is a traditional support system for perinatal women, called “*satogaeri-bunben*,” in which pregnant women return to their home town or family home for delivery and postpartum rest (39). About half the women answered they went back to their parents' home until the child is 1 month old (38). Even if they did not choose this system, “reversed *satogaeri-bunben*,” in which grandmothers joined the family for support, is common (40). Because of these Japanese cultural backgrounds, the relationship with parents is assumed to be important for psychological wellbeing of postpartum women.

We hypothesized that in addition to consistently unsatisfied parent-daughter relationship, deteriorating relationship was also associated with PPD. Thus, using a hospital-based sample, we attempted to address whether the trajectories of parent-daughter relationship during pregnancy relate to PPD in Japan.

## Methods

### Participants

**Figure 1:**
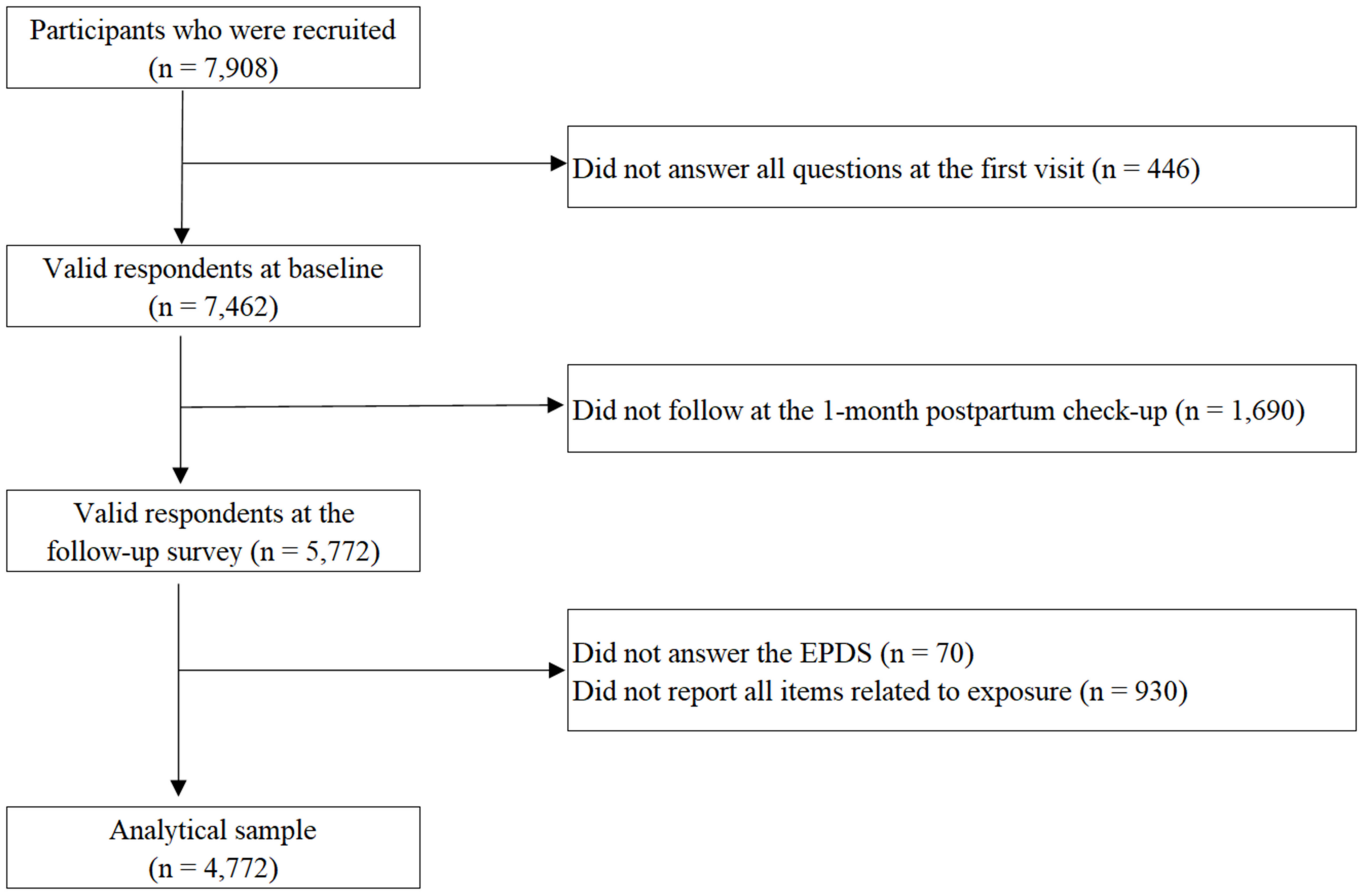
Flow chart of participants.

This is a hospital-based prospective cohort study. Participants were recruited at maternity hospitals, that agreed to participate, in four prefectures covering the east (Miyagi prefecture) and west regions (Osaka, Kagawa, and Oita prefectures) in Japan [58 out of 214 delivery facilities in the regions (41)]. These hospitals range from urban perinatal center to local maternity facility. The target subjects were all women who visited the participating facilities for their delivery between April 2019 and March 2020. Participants were asked to answer the questionnaire including parent-daughter relationship at their first visit and after delivery within 1-week. Participants were then followed up on mental health at their 1-month postpartum check-up. Written informed consent was obtained from all study subjects. A baseline questionnaire was distributed to 7,908 women. There were 7,462 who answered the baseline questionnaire with at least one response (response rate: 94.4%) and 5,772 who answered the questionnaire at the follow-up survey at 1-month postpartum (follow-up rate: 77.4%). Among the valid responses, we excluded the participants with missed exposure or outcome variables in this study [i.e., relationship with their parents at baseline or postpartum (*n* = 930), and PPD (*n* = 70)]. The final analytical sample included 4,772 women (Figure 1). The participants who were excluded due to missing data of variables did not show significant differences in maternal age group, history of psychiatric disorder, economic status, feelings at pregnancy, quarrel with partner, marital status, maternal education, and parity compared with the analytical sample (all *p* > 0.05, Supplementary material 1). This study was approved by the institutional review boards of Osaka Women's and Children's Hospital.

### Measurement

PPD was assessed by the Edinburgh Postnatal Depression Scale (EPDS) (42). It consists of 10 questions, each of which is scored from 0 to 3 points, for a total score of 0 to 30 points. The validity and reliability of the Japanese version of EPDS was previously reported (43). Using a cutoff of 8/9 points, sensitivity and specificity in Japanese postpartum women were reported to be 75 and 93%, respectively (43). In this study, PPD was determined at a score of 9 or more on the EPDS at 1-month postpartum check-up.

Parent-daughter relationship was asked by the following question: “Are you satisfied with the relationship you have with your parents? Please circle each of the following: satisfied, not very satisfied, not satisfied at all.” Pregnant women of satisfied parent-daughter relationship at both baseline and postpartum was defined as “consistently satisfied.” Likewise, unsatisfied relationship at baseline and satisfied relationship at postpartum, satisfied relationship at baseline and unsatisfied relationship at postpartum, or unsatisfied relationship at both time points were defined as “improving,” “deteriorating,” and “consistently unsatisfied,” respectively. As for criterion validity, the question was compared with social support status (whether there is someone you can talk to when you need help), based on previous literature showing that parent-child relationship determined social support in adulthood (44). We confirmed that a sensitivity of 93.9% and a specificity of 75% in identifying women with no support from others when answers of “not very satisfied” and “not satisfied at all” were defined as abnormal.

As covariates, maternal characteristics were assessed at baseline. That is, maternal age group, history of psychiatric disorder (45) (“never,” “past,” “current”), economic status (46) (“stable,” “not so stable,” “unstable”), feelings when pregnancy was confirmed (46) (“happy,” “unexpected but happy,” “unexpected and confused,” “did not know what to do,” “no feelings”), frequency of quarrel with partner (46) (“none,” “sometimes,” “often”), marital status (11) (“married,” “plan to get married,” “unmarried,” “remarried taking one's child”), and education (47) (“high school or more,” “retirement from high school,” “junior high school”) were assessed with a baseline questionnaire. We also assessed perinatal characteristics including child's sex (“male,” “female”), first child (48) (“Yes,” “No”), child's birth weight (“ <1,500g,” “1,500–2,499 g,” “≥2,500 g”), premature birth (“yes,” “no”), and modes of delivery (“vaginal delivery,” “labor analgesia,” “planned cesarean delivery,” “emergent cesarean delivery”).

### Statistical analysis

Multivariable binary logistic regression model was applied to adjust for covariates. Model 1 was adjusted for maternal age group. Model 2 was further adjusted for history of psychiatric disorder as a potential confounder. Model 3 was adjusted for covariates in model 2 plus maternal and perinatal characteristics (economic status, feelings at pregnancy, quarrel with partner, marital status, education, first child, and residential prefecture). We treated missing values in covariates as dummy variables. A *p*-value of < 0.05 was considered statistically significant. All analyses were conducted using STATA MP version 16.0 (STATA Corporation, College Station, TX, USA).

## Results

Table 1 shows the distribution of characteristics of analytic sample. The number of participants in Oita, Osaka, Miyagi, and Kagawa prefecture was 2,488 (52.1%), 1,329 (27.9%), 897 (18.8%), and 58 (1.2%), respectively. The median of the first assessment was 11 (interquartile range 9–17). The number of women with consistently satisfied, improving, deteriorating, and consistently unsatisfied relationship with parents was 4,340 (91.0%), 129 (2.7%), 122 (2.6%), and 181 (3.8%), respectively. The distribution of parent-daughter relationship at two assessment points were reported in Supplementary material 2. Approximately 6% of women had previously been treated or currently being treated for mental illness, 3% did not feel happy when pregnancy was confirmed, and 3% reported unstable family finances. Women other than those in the consistently satisfied group tended to have past psychiatric disorders. Regarding perinatal characteristics, 41% were first-time mothers, 8% had a low or extremely low birthweight baby, 4% had preterm labor, 3% delivered with labor analgesia, and 19% had planned or emergent cesarean section.

**Table 1:** Descriptive characteristics of the study participants at baseline.

	**The trajectory of the parent-daughter relationship**							
	**Total** **(*****n*** = **4,772)**		**Consistently satisfied** **(*****n*** = **4,340; 91.0%)**		**Improving** **(*****n*** = **129; 2.7%)**		**Deteriorating** **(*****n*** = **122; 2.6%)**		**Consistently unsatisfied** **(*****n*** = **181; 3.8%)**	
	***n***	**%**	***n***	**%**	***n***	**%**	***n***	**%**	***n***	**%**
**Gestational week of the first assessment (median)**	11	IQR 9–17	11	IQR 9–17	11	IQR 9–16	11	IQR 9–16	12	IQR 10–19
**Maternal age (years)**										
<25	414	8.7	383	8.8	11	8.5	5	4.1	15	8.3
25–29	1,211	25.4	1,120	25.8	26	20.2	29	23.8	36	19.9
30–34	1,705	35.7	1,546	35.6	53	41.1	48	39.3	58	32.0
35–39	1,113	23.3	1,002	23.1	31	24.0	29	23.8	51	28.2
≥40	274	5.7	247	5.7	2	1.6	7	5.7	18	9.9
**History of psychiatric disorders**										
Missing	55	1.2	42	1.0	4	4.7	4	3.3	3	1.7
Never	4,512	94.6	4,148	95.6	116	89.9	105	86.1	143	79.0
Past	198	4.2	145	3.3	10	7.8	12	9.8	31	17.1
Current	58	1.2	43	1.0	3	2.3	5	4.1	7	3.9
Missing	4	0.1	4	0.1	0	0.0	0	0.0	0	0.0
**Economic status**										
Stable	2,804	58.8	2,618	60.3	53	41.1	58	47.5	75	41.4
Not so stable	1,807	37.9	1,605	37.0	60	46.5	58	47.5	84	46.4
Unstable	155	3.3	111	2.6	16	12.4	6	4.9	22	12.2
Missing	6	0.1	6	0.1	0	0.0	0	0.0	0	0.0
**Feelings at pregnancy**										
Happy	3,449	72.3	3,180	73.3	81	62.8	81	66.4	107	59.1
Unexpected but happy	1,175	24.6	1,034	23.8	44	34.1	37	30.3	60	33.2
Unexpected and confused/Did not know what to do/No feelings	146	3.1	124	2.9	4	3.1	4	3.3	14	7.7
Missing	2	0.0	2	0.1	0	0.0	0	0.0	0	0.0
**Quarrel with partner**										
None	2,460	51.6	2,285	52.7	56	43.4	50	41.0	69	38.1
Sometimes	2,179	45.7	1,950	44.9	63	48.8	68	55.7	98	54.1
Often	119	2.5	95	2.2	9	7.0	3	2.5	12	6.6
Missing	14	0.3	10	0.2	1	0.8	1	0.8	2	1.1
**Marital status**										
Married	4,321	90.6	3,958	91.2	104	80.6	107	87.7	152	84.0
Plan to get married	307	6.4	271	6.2	10	7.8	10	8.2	16	8.8
Unmarried/Remarried taking one's child	139	2.9	108	2.5	14	10.9	4	3.3	13	7.2
Missing	5	0.1	3	0.1	1	0.8	1	0.8	0	0.0
**Maternal education**										
High school or more	4,568	95.7	4,173	96.2	117	90.7	114	93.4	164	90.6
Retirement from high school	129	2.7	108	2.5	7	5.4	6	4.9	8	4.4
Junior high school	74	1.6	58	1.3	5	3.9	2	1.6	9	5.0
Missing	1	0.0	1	0.0	0	0.0	0	0.0	0	0.0
**Prefecture of the study institution**										
Osaka	1,329	27.9	1,231	28.4	25	19.4	36	29.5	37	20.4
Miyagi	897	18.8	804	18.5	31	24.0	23	18.9	39	21.6
Kagawa	58	1.2	49	1.1	5	3.9	2	1.6	2	1.1
Oita	2,488	52.1	2,256	52.0	68	52.7	61	50.0	103	56.9
**Number of people to consult**										
Several	4,575	95.9	4,217	97.2	106	82.2	112	91.8	140	77.4
Only one	186	3.9	120	2.8	20	15.5	8	6.6	38	21.0
None	10	0.2	2	0.1	3	2.3	2	1.6	3	1.7
Missing	1	0.0	1	0.0	0	0.0	0	0.0	0	0.0
**Child's sex**										
Male	2,410	50.5	2,200	50.7	64	49.6	61	50.0	85	47.0
Female	2,288	48.0	2,072	47.7	63	48.8	59	48.4	94	51.9
Missing	74	1.6	68	1.6	2	1.6	2	1.6	2	1.1
**First child**										
Yes	1,955	41.0	1,788	41.2	45	34.9	53	43.4	69	38.1
No	2,732	57.3	2,474	57.0	82	63.6	67	54.9	109	60.2
Missing	85	1.8	78	1.8	2	1.6	2	1.6	3	1.7
**Child's birth weight (gram)**										
<1,500	15	0.3	12	0.3	1	0.8	1	0.8	1	0.6
1,500–2,499	355	7.4	317	7.3	10	7.8	11	9.0	17	9.4
2,500	4,297	90.1	3,914	90.2	116	89.9	108	88.5	159	87.9
Missing	105	2.2	97	2.2	2	1.6	2	1.6	4	2.2
**Premature birth**										
Yes	190	4.0	170	3.9	6	4.7	6	4.9	8	4.4
No	4,506	94.4	4,100	94.5	121	93.8	114	93.4	171	94.5
Missing	76	1.6	70	1.6	2	1.6	2	1.6	2	1.1
**Modes of delivery**										
Vaginal delivery	3,646	76.4	3,333	76.8	94	72.9	88	72.1	131	72.4
Labor analgesia	153	3.2	138	3.2	10	7.8	2	1.6	3	1.7
Planned cesarean delivery	598	12.5	529	12.2	16	12.4	20	16.4	33	18.2
Emergent cesarean delivery	305	6.4	276	6.4	7	5.4	10	8.2	12	6.6
Missing	70	1.5	64	1.5	2	1.6	2	1.6	2	1.1

Values are presented as frequencies with percentages unless specified.

Table 2 shows the results of the logistic regression analyses to examine the association between relationship trajectories of pregnant women with their parents and PPD. In the age-adjusted model (Model 1), compared to the consistently satisfied group, pregnant women with improving, deteriorating, and consistently unsatisfied group showed 1.78 [95% confidence interval (CI): 1.04–3.05], 3.24 (95% CI: 2.05–5.11), 3.37 (95% CI: 2.31–4.92) times more likely to show PPD, respectively. When a history of psychiatric disorders was added, pregnant women with improving, deteriorating, and consistently unsatisfied group was 1.61 (95% CI: 0.93–2.79), 2.82 (95% CI: 1.77–4.51), and 2.66 (95% CI: 1.80–3.94) times more likely to show PPD (Model 2). After controlling for other possible confounders in Model 3, pregnant women with deteriorating and consistently unsatisfied group were 2.81 (95% CI: 1.73–4.55) and 2.39 (95% CI: 1.58–3.62) times more likely to have PPD. The improving relationship group did not show statistically significant risk of PPD [odds ratio (OR): 1.50; 95% CI: 0.84–2.68]. When comparing the deteriorating group and the consistently unsatisfied group, there was no statistically significant difference (OR: 1.13; 95% CI: 0.62–2.04).

**Table 2:** Adjusted odds ratio with 95% confidence intervals for the association of the trajectory of parent-daughter relationship and postpartum depression in Japanese women.

	**Model 1**	**Model 2**	**Model 3**
	**OR (95% CI)**	**OR (95% CI)**	**OR (95% CI)**
**The trajectory of parent-daughter relationship**			
Consistently satisfied	Ref	Ref	Ref
Improving	**1.78 (1.04–3.05)**	1.61 (0.93–2.79)	1.50 (0.84–2.68)
Deteriorating	**3.24 (2.05–5.11)**	**2.82 (1.77–4.51)**	**2.81 (1.73–4.55)**
Consistently unsatisfied	**3.37 (2.31–4.92)**	**2.66 (1.80–3.94)**	**2.39 (1.58–3.62)**
**History of psychiatric disorders**			
Never		Ref	Ref
Past		**3.41 (2.39–4.88)**	**3.12 (2.15–4.53)**
Current		**4.52 (2.50–8.17)**	**4.09 (2.16–7.75)**
**First child**			
Yes			Ref
No			**0.29 (0.23–0.37)**
**Quarrel with partner**			
None			Ref
Sometimes			1.20 (0.96–1.51)
Often			**2.72 (1.62–4.57)**
**Financial status**			
Stable			Ref
Not so stable			**1.46 (1.16–1.84)**
Unstable			**2.31 (1.38–3.85)**
**Feelings at pregnancy**			
Happy			Ref
Unexpected but happy			1.25 (0.98–1.61)
Unexpected and confused/Did not know what to do/No feelings			0.96 (0.51–1.81)
**Marital status**			
Married			Ref
Plan to get married			0.91 (0.59–1.38)
Unmarried/Remarried taking one's child			0.58 (0.28–1.21)
**Education**			
High school or more			Ref
Retirement from high school			**2.04 (1.13–3.70)**
Junior high school			0.73 (0.29–1.87)
**Prefecture**			
Osaka			Ref
Miyagi			0.77 (0.54–1.12)
Kagawa			1.11 (0.41–3.00)
Oita			**1.40 (1.08–1.82)**

Values are odds ratios with 95% confidence intervals given in parentheses estimated with multivariable binary logistic regression analyses.

Model 1: Adjusted for age group.

Model 2: Model 1 + adjusted for history of psychiatric disorders.

Model 3: Model 2 + adjusted for pregnancy demographics (first child, economic status, feelings at pregnancy, quarrel with partner, marital status, education, and prefecture).

Bold values mean statistically significant.

## Discussion

To the best of our knowledge, this is the first study to show that deterioration of parent-daughter relationship in the pre and postpartum period, as perceived by pregnant women, was associated with PPD using prospective hospital-based study in Japan. This association persists after adjusting for possible confounding factors, such as history of psychiatric disorders, financial status, or feelings at pregnancy, suggesting that deterioration of parent-daughter relationship is independently associated with PPD. In contrast, women with improving parent-daughter relationship were not at risk of PPD after adjusting for confounders.

The result of this study is partially consistent with previous literature. A clinic-based cohort study in the UK of 119 primipara, assessing their mother-daughter relationship at 12–14 gestational weeks, found that those who had problems with the relationship with their mother at early pregnancy were more likely develop PPD. In the study, however, the trajectories of mother-daughter relationship was not assessed (23). As we had originally hypothesized, we have added new evidence that PPD is associated with deteriorating and consistently unsatisfied parent-daughter relationship during pregnancy among Japanese parturient women with a longitudinal assessment of the relationship.

There are two possible explanations of how deterioration of parent-daughter relationship induced PPD. First, deteriorating parent-daughter relationship lessened the emotional and instrumental support from parents, the main support providers for postpartum women in Japan (49), and the lack of social support from them induced PPD (50, 51). Second, deteriorating parent-daughter relationship may induce stress and trigger an inflammatory response during pregnancy, which can be another cause of PPD. Parents (especially mothers) and daughters tend to reevaluate each other during pregnancy with the daughters' transitions to motherhood and parents' aging and infirmity (52). They have different “developmental stakes”; daughters seek to establish their independence while parents strive for a sense of continuity and connectedness with their daughters (52, 53). This discrepancy could be a source of mental stress for both. Stress is also added due to the different value on how children should be raised in different generations (54). Because we found no association between improving parent-daughter relationship group, rather than the stress in early pregnancy, stress in later pregnancy due to parent-daughter relationship has an impact on PPD, which is probably attributable to epigenetic and neuroendocrine changes in later pregnancy (55).

We also found a robust association between consistently unsatisfied parent-daughter relationship and PPD, which can be explained by the following pathways. First, women with consistently unsatisfied parent-daughter relationship may have insecure attachment style, which is associated with deficits in psychological resilience and adaptation to stress, thus rendering the women vulnerable to life transitions at postpartum (56). In fact, adults who were maltreated by their parents as children continue to experience challenges in the parent-daughter relationship (17). Women with poor parent-daughter relationship at postpartum may find it difficult to mitigate the negative emotions they experience while raising children. In other words, intimacy with parents provides the foundation for psychological stability for postpartum women (57). Second, women with poor parent-daughter relationship may not choose “*satogaeri-bunben*” and/or they will not seek support from their parents, leading them to be at risk of PPD. In modern society where regional communities are diminishing and social child rearing support services are inadequate, support from parents including “s*atogaeri-bunben*” has been reported to decrease PPD because it (1) enables parturient to rest, (2) resolves their anxiety, and (3) provides an opportunity to learn how to care for babies (58). However, a stable parent-daughter relationship may be a prerequisite to ask for parental support or to stay at their family home for a few months (59). Women without adequate support from parents due to unsatisfied parent-daughter relationship may suffer from physical fatigue and anxiety. Further study is needed to clarify the mechanism between the trajectories of parent-daughter relationship and PPD.

The present study has several limitations. First, the sample may not be representative of the whole population because we collected data from only four prefectures in Japan. Accordingly, the findings of this study may not be generalized. Second, we used EPDS to measure the outcome. While it is a validated screening tool, it is not the clinical criteria for PPD. Third, parent-daughter relationship was solely assessed using the originally created question on whether women are satisfied with their parents. This question is based on the theory of internal working models (60, 61), in which women of secure type were characterized by trust and emotional satisfaction (62). Although we adopted this question for simplicity in clinical practice, future studies may use validated scales (i.e., Prenatal Self-Evaluation Questionnaire) (63). Fourth, we assessed overall satisfaction with the relationship with their parents, and we could not distinguish whether women with unsatisfied parent-daughter relationship had a history of childhood abuse or not. To account for this limitation, a more detailed question on childhood abuse is necessary. However, the feasibility of such questionnaire is uncertain in the clinical setting because participants may feel distressed, even momentarily, when recalling their uncomfortable or traumatic experience or memories (64). Fifth, other known risk factors such as genetic vulnerabilities and stressful life events were not assessed in this study.

Despite these limitations, the current finding provides new insights into the screening of women at high risk of PPD. Although midwives and obstetricians do ask pregnant women about their risk factors for PPD at initial assessment, including parent-daughter relationship, most of them do not reevaluate these risks at postpartum, assuming that such characteristics may not change during pregnancy. In order to detect pregnant women at risk of PPD, health care providers need to take advantage of the opportunity to talk regularly with pregnant women and pay attention to the trajectories in relationship during pregnancy. Furthermore, interventions to improve parent-daughter relationship during pregnancy may be beneficial as we showed that pregnant women with improving relationship with their parents showed a similar risk as women of consistently satisfactory relationship with parents. Midwives and obstetricians could contribute to promoting positive change in the parent-daughter relationship, for example, by suggesting daughters to listen to their mothers' birth experiences or informing parents about what types of support would be most beneficial to their daughters after childbirth (32). Further intervention studies improving parent-daughter relationship by health care providers are warranted.

In conclusion, pregnant women who felt that their relationship with parents “deteriorated” or was “consistently unsatisfactory” during pregnancy showed increased risk for PPD. These findings can be applied to develop more effective screening method of PPD with the goal of decreasing the victims of “a dangerous thief.”

## Data availability statement

The datasets generated for this study are not publicly available because the Ethics committee did not give permission for the data to be made publicly available. Further inquiries can be directed to the corresponding author/s.

## Ethics statement

The studies involving human participants were reviewed and approved by the Institutional Review Boards of Osaka Women's and Children's Hospital. The patients/participants provided their written informed consent to participate in this study.

## Author contributions

ST: visualization and writing—original draft preparation. SD, YT, YM, and AI: visualization, data curation, and writing—review and editing. TF: project administration, supervision, and writing—review and editing. JS, KM, SS, and NM: conceptualization and investigation. All authors have approved the final manuscript.

## Funding

This study was partially supported by the Ministry of Health, Labor and Welfare (H27-Sukoyaka-Ippan-001, H30-Sukoyaka-Ippan-003, and 21DA1004).

## Conflict of interest

The authors declare that the research was conducted in the absence of any commercial or financial relationships that could be construed as a potential conflict of interest.

## Publisher's note

All claims expressed in this article are solely those of the authors and do not necessarily represent those of their affiliated organizations, or those of the publisher, the editors and the reviewers. Any product that may be evaluated in this article, or claim that may be made by its manufacturer, is not guaranteed or endorsed by the publisher.

## References

[1] O'Hara MW McCabe JE. Postpartum depression: current status and future directions. Annu Rev Clin Psychol. (2013) 9:379–407. 10.1146/annurev-clinpsy-050212-185612 23394227

[2] Shorey S Chee CYI Ng ED Chan YH Tam WWS Chong YS. Prevalence and incidence of postpartum depression among healthy mothers: a systematic review and meta-analysis. J Psychiatr Res. (2018) 104:235–48. 10.1016/j.jpsychires.2018.08.001 30114665

[3] Hutchens BF Kearney J. Risk factors for postpartum depression: an umbrella review. J Midwifery Womens Health. (2020) 65:96–108. 10.1111/jmwh.13067 31970924

[4] Beck CT. Postpartum depression stopping the thief that steals motherhood AWHONN lifelines. Nurs Women's Health. (1999) 3:41–4. 10.1111/j.1552-6356.1999.tb01115.x 10690011

[5] Weng SC Chang JC Yeh MK Wang SM Chen YH. Factors influencing attempted and completed suicide in postnatal women: a population-based study in Taiwan. Sci Rep. (2016) 6:25770. 10.1038/srep25770 27173845 PMC4865942

[6] Krischer MK Stone MH Sevecke K Steinmeyer EM. Motives for maternal filicide: results from a study with female forensic patients. Int J Law Psychiatry. (2007) 30:191–200. 10.1016/j.ijlp.2007.03.003 17449099

[7] Buist A. Childhood abuse, parenting and postpartum depression. Aust N Z J Psychiatry. (1998) 32:479–87. 10.3109/00048679809068320 9711360

[8] Dennis C-L Ross LE. Depressive symptomatology in the immediate postnatal period: identifying maternal characteristics related to true-and false-positive screening scores. Can J Psychiatry. (2006) 51:265–73. 10.1177/070674370605100501 16986815

[9] Garabedian MJ Lain KY Hansen WF Garcia LS Williams CM Crofford LJ. Violence against women and postpartum depression. J Womens Health. (2011) 20:447–53. 10.1089/jwh.2010.1960 21323583

[10] Lang AJ Rodgers CS Lebeck MM. Associations between maternal childhood maltreatment and psychopathology and aggression during pregnancy and postpartum. Child Abuse Negl. (2006) 30:17–25. 10.1016/j.chiabu.2005.07.006 16406025

[11] Plaza A Garcia-Esteve L Torres A Ascaso C Gelabert E Luisa Imaz M. Childhood physical abuse as a common risk factor for depression and thyroid dysfunction in the earlier postpartum. Psychiatry Res. (2012) 200:329–35. 10.1016/j.psychres.2012.06.032 22878032

[12] Ehrensing RH Weitzman EL. The mother-daughter relationship in anorexia nervosa. Psychosom Med. (1970) 32:201–8. 10.1097/00006842-197003000-00006 5265902

[13] Yamanaka C Kawata K. Characteristics of mother-daughter relationships and sexual risk-coping consciousness among Japanese Female University Students. Int J Environ Res Public Health. (2020) 17:8795. 10.3390/ijerph17238795 33256149 PMC7730645

[14] Samari G Seltzer JA. Risky sexual behavior of foreign and native-born women in emerging adulthood: the long reach of mother-daughter relationships in adolescence. Soc Sci Res. (2016) 60:222–35. 10.1016/j.ssresearch.2016.06.003 27712681 PMC5116325

[15] Cederbaum JA Adhikari AB Guerrero EG Hutchinson MK. Relationship satisfaction and communication among urban minority HIV-positive and Hiv-negative mothers: the influence on daughter's alcohol use. J Fam Issues. (2016) 37:155–76. 10.1177/0192513X13513582 26900198 PMC4758986

[16] Zayas LH Hausmann-Stabile C Kuhlberg J. Can better mother-daughter relations reduce the chance of a suicide attempt among latinas? Depress Res Treat. (2011) 2011:403602. 10.1155/2011/403602 21822487 PMC3148596

[17] Kong J Martire LM. Parental childhood maltreatment and the later-life relationship with parents. Psychol Aging. (2019) 34:900–11. 10.1037/pag0000388 31478703 PMC6958553

[18] Shawler C Edward J Ling J Crawford TN Rayens MK. Impact of mother-daughter relationship on hypertension self-management and quality of life: testing dyadic dynamics using the actor-partner interdependence model. J Cardiovasc Nurs. (2018) 33:232–8. 10.1097/JCN.0000000000000448 28990970 PMC5886818

[19] Flynn SJ Ameling JM Hill-Briggs F Wolff JL Bone LR Levine DM. Facilitators and barriers to hypertension self-management in urban African Americans: perspectives of patients and family members. Patient Prefer Adherence. (2013) 7:741–9. 10.2147/PPA.S46517 23966772 PMC3743518

[20] Antonucci TC Ajrouch KJ Birditt KS. The convoy model: explaining social relations from a multidisciplinary perspective. Gerontologist. (2014) 54:82–92. 10.1093/geront/gnt118 24142914 PMC3894851

[21] Bengtson VL. Beyond the nuclear family: the increasing importance of multigenerational bonds: the burgess award lecture. J Marriage Fam. (2001) 63:1–16. 10.1111/j.1741-3737.2001.00001.x

[22] Boyce P Hickie I Parker G. Parents, partners or personality? Risk factors for post-natal depression. J Affect Disord. (1991) 21:245–55. 10.1016/0165-0327(91)90004-C 1829746

[23] Kumar R Robson KM. A prospective study of emotional disorders in childbearing women. Br J Psychiatry. (1984) 144:35–47. 10.1192/bjp.144.1.35 6692075

[24] O'Hara MW Rehm LP Campbell SB. Predicting depressive symptomatology: cognitive-behavioral models and postpartum depression. J Abnorm Psychol. (1982) 91:457–61. 10.1037/0021-843X.91.6.457 7153419

[25] Tambelli R Trentini C Trovato A Volpi B. Role of psychosocial risk factors in predicting maternal and paternal depressive symptomatology during pregnancy. Infant Ment Health J. (2019) 40:541–56. 10.1002/imhj.21791 31062378

[26] El Saied HF Eissa A El Kholy TS. A Cross-sectional study of risk factors for postpartum depression. Egypt J Psychiatry. (2021) 42:108. 10.4103/ejpsy.ejpsy_10_17

[27] Smorti M Ponti L Pancetti F. A comprehensive analysis of post-partum depression risk factors: the role of socio-demographic, individual, relational, and delivery characteristics. Front. Public Health. (2019) 7:295. 10.3389/fpubh.2019.00295 31709213 PMC6821715

[28] Pilkington PD Milne LC Cairns KE Lewis J Whelan TA. Modifiable partner factors associated with perinatal depression and anxiety: a systematic review and meta-analysis. J Affect Disord. (2015) 178:165–80. 10.1016/j.jad.2015.02.023 25837550

[29] Tilden VP. A developmental conceptual framework for the maturational crisis of pregnancy. West J Nurs Res. (1980) 2:667–78. 10.1177/019394598000200402

[30] Ballou J. The significance of reconciliative themes in the psychology of pregnancy. Bull Menninger Clin. (1978) 42:383. 719211

[31] Lederman RP Weis KL. Relationship with Mother. Psychosocial Adaptation to Pregnancy. Berlin: Springer (2020). p. 105-21.

[32] Martell LK. The mother-daughter relationship during daughter's first pregnancy: the transition experience. Holist Nurs Pract. (1990) 4:47–55. 10.1097/00004650-199005000-00008 2329137

[33] Nagatsuru M Takahashi M. Changes in the mother-daughter relationship from pregnancy. J Jpn Soc Nurs Health Care. (2002) 4:11–7. Available online at: https://cir.nii.ac.jp/crid/1050282812550256896

[34] Stern G Kruckman L. Multi-disciplinary perspectives on post-partum depression: an anthropological critique. Soc Sci Med. (1983) 17:1027–41. 10.1016/0277-9536(83)90408-2 6623110

[35] Withers M Kharazmi N Lim E. Traditional beliefs and practices in pregnancy, childbirth and postpartum: a review of the evidence from Asian countries. Midwifery. (2018) 56:158–70. 10.1016/j.midw.2017.10.019 29132060

[36] Ministry of Health Labour and Welfare. Status of Male Employees Taking Childcare Leave and Efforts to Encourage Them to Take Such Leave. (2019). Available online at: https://www8.cao.go.jp/shoushi/shoushika/meeting/consortium/04/pdf/houkoku-2.pdf (accessed May 21, 2021).

[37] Ministry of Health Labour and Welfare. Report on the Survey Research Project on the Actual Conditions of Users of Postpartum Care Services. (2020). Available online at: https://www.mhlw.go.jp/content/000694012.pdf (accessed May 21, 2021).

[38] Benesse Educational Research Development Institute. Survey on Prenatal and Postnatal Support for Mothers in Japan. (2015). Available online at: https://berd.benesse.jp/up_images/research/sanzensango.pdf (accessed May 20, 2021).

[39] Shinagawa N. Social and medical discussion on Satogaeri Bunben. Nihonishikai Zasshi. (1978) 80:351–5.11285079

[40] Yoshida K Yamashita H Ueda M Tashiro N. Postnatal depression in Japanese mothers and the reconsideration of 'Satogaeri Bunben'. Pediatr Int. (2001) 43:189–93. 10.1046/j.1442-200x.2001.01370.x 11285079

[41] Committee for Establishing a Sustainable Obstetrics and Gynecology Medical System, Japanese Society of Obstetrics and Gynecology. Childbirth Facility Search. Available online at: https://shusanki.org/area.html (accessed July 09, 2021).

[42] Cox JL Holden JM Sagovsky R. Detection of postnatal depression: development of the 10-item Edinburgh Postnatal Depression Scale. Br J Psychiatry. (1987) 150:782–6. 10.1192/bjp.150.6.782 3651732

[43] Okano T. Validation and reliability of a Japanese version of the EPDS. Arch Psychiatr Diagn Clin Eval. (1996) 7:525–33.

[44] Okayama H. Developing and analyzing the reliabioity and validity of a primigravida-mother relationship scale. J Jpn Acad Nurs Sci. (2011) 31:3–13. 10.5630/jans.31.1_3

[45] Milgrom J Gemmill AW Bilszta JL Hayes B Barnett B Brooks J. Antenatal risk factors for postnatal depression: a large prospective study. J Affect Disord. (2008) 108:147–57. 10.1016/j.jad.2007.10.014 18067974

[46] Yamada A Isumi A Fujiwara T. Association between lack of social support from partner or others and postpartum depression among Japanese Mothers: a population-based cross-sectional study. Int J Environ Res Public Health. (2020) 17:4270. 10.3390/ijerph17124270 32549294 PMC7345875

[47] Li Y Long Z Cao D Cao F. Social support and depression across the perinatal period: a longitudinal study. J Clin Nurs. (2017) 26:2776–83. 10.1111/jocn.13817 28334472

[48] Hetherington E McDonald S Williamson T Tough S. Trajectories of social support in pregnancy and early postpartum: findings from the all our families cohort. Soc Psychiatry Psychiatr Epidemiol. (2020) 55:259–67. 10.1007/s00127-019-01740-8 31256206

[49] Kita A. Characteristics of the social support for pregnant women in Japan: according to concept of four kinds of social support. J Jpn Acad Nurs Sci. (1997) 17:8–21. 10.5630/jans1981.17.1_8 9274369

[50] Kim TH Connolly JA Tamim H. The effect of social support around pregnancy on postpartum depression among Canadian teen mothers and adult mothers in the maternity experiences survey. BMC Pregnancy Childbirth. (2014) 14:162. 10.1186/1471-2393-14-162 24884410 PMC4018615

[51] Chen HH Hwang FM Lin LJ Han KC Lin CL Chien LY. Depression and social support trajectories during 1 year postpartum among marriage-based immigrant mothers in Taiwan. Arch Psychiatr Nurs. (2016) 30:350–5. 10.1016/j.apnu.2015.12.008 27256940

[52] Fischer LR. Transitions in the mother-daughter relationship. J Marriage Family. (1981) 1981:613–22. 10.2307/351762

[53] Bengtson VL Kuypers JA. Generational difference and the developmental stake. Aging Human Dev. (1971) 2:249–60. 10.2190/AG.2.4.b

[54] Iseki A Shirai M. The feeling within experiences supported by their mothers for nursing and child caring, and related factors. Maternal Health. (2010) 50:672–9.31166199

[55] Payne JL Maguire J. Pathophysiological mechanisms implicated in postpartum depression. Front Neuroendocrinol. (2019) 52:165–80. 10.1016/j.yfrne.2018.12.001 30552910 PMC6370514

[56] Allen JG Fonagy P Bateman AW. Mentalizing in Clinical Practice. Washington, DC: American Psychiatric Publication (2008).

[57] Kitamura K Takashi M. The influence of adult mother-daughter relationships on daughters' psychological well-being: life events of marriage and childbearing. Jpn J Dev Psychol. (2001) 12:46–57. 10.11201/jjdp.12.46

[58] Okano T Nomura J Koshikawa N. Cross-cultural study of maternity blues and postpartum depression. Seishin Igaku. (1991) 33:1051–8.

[59] Kobayashi Y. Assistance received from parturients' own mothers during 'Satogaeri' (their perinatal visit and stay with their parents) and development of the mother-infant relationship and maternal identity. J Jpn Acad Midwif. (2010) 24:28–39. 10.3418/jjam.24.28

[60] Bowlby J. Attachment and loss: Volume II: Separation, Anxiety and Anger. London: The Hogarth Press and the Institute of Psycho-analysis (1973). p. 1–429.

[61] Muller ME. Development of the prenatal attachment inventory. West J Nurs Res. (1993) 15:199–211; discussion 5. 10.1177/019394599301500205 8470375

[62] Hazen C Shaver P. Romantic love conceptualized as an attachment process. J Pers Soc Psychol. (1987) 52:511–24. 10.1037/0022-3514.52.3.511 3572722

[63] Lederman R Weis K. Psychosocial Adaptation in Pregnancy: Assessment of Seven Dimensions of Maternal Development. Berlin: Springer (2009). p. 1–38. 10.1007/978-1-4419-0288-7_1

[64] Allmark P Boote J Chambers E Clarke A McDonnell A Thompson A. Ethical issues in the use of in-depth interviews: literature review and discussion. Res Ethics. (2009) 5:48–54. 10.1177/174701610900500203

