# Supplementary material for: Relationship trajectories of pregnant women with their parents and postpartum depression: A hospital-based prospective cohort study in Japan
Source: Front Psychiatry. 2022 Nov 3;13:961707. doi: 10.3389/fpsyt.2022.961707 (PMC9668856; doi:10.3389/fpsyt.2022.961707)
Supplement: Supplementary file 1 [file Data_Sheet_1.docx]

| Supplementary Table 1. Characteristics between analytical and excluded samples. | | | |  |
| --- | --- | --- | --- | --- |
|  |  | Analytical sample  (*n* = 4,772) | Excluded sample  (*n* = 3,136) |  |
|  |  | % | % | *p* value |
| Age group | < 25 | 8.8 | 7.7 |  |
|  | 25 – 29 | 25.7 | 25.1 |  |
|  | 30 – 34 | 36.2 | 36.7 |  |
|  | 35 – 39 | 23.6 | 24.1 |  |
|  | 40+ | 5.8 | 6.4 | 0.815 |
| History of psychiatric disorder | Never | 94.6 | 93.9 |  |
|  | Past | 4.2 | 4.4 |  |
|  | Current | 1.2 | 1.7 | 0.186 |
| Economic status | Stable | 58.8 | 59.2 |  |
|  | Not so stable | 37.9 | 37.4 |  |
|  | Unstable | 3.3 | 3.4 | 0.904 |
| Feelings at pregnancy | Happy | 72.3 | 73.0 |  |
|  | Unexpected but happy | 24.6 | 23.4 |  |
|  | Unexpected and confused /Did not know what to do /No feelings | 3.1 | 3.3 | 0.235 |
| Quarrel with partner | None | 51.7 | 50.8 |  |
|  | Sometimes | 45.8 | 47.1 |  |
|  | Often | 2.5 | 2.1 | 0.372 |
| Marital status | Married | 90.6 | 89.4 |  |
|  | Plan to get married | 6.4 | 7.3 |  |
|  | Unmarried/ Remarried taking one's child | 2.9 | 3.3 | 0.196 |
| Education | High school or more | 95.8 | 95.5 |  |
|  | Retirement from high school | 2.7 | 2.8 |  |
|  | Junior high school | 1.6 | 1.8 | 0.738 |
| Parity | Primipara | 41.7 | 43.1 |  |
|  | Multipara | 57.3 | 22.9 | 0.393 |
| *p*-value is based on chi-square test. | |  |  |  |

| Supplementary Table 2. The distribution of the parent-daughter relationship at two assessment points | | | | |
| --- | --- | --- | --- | --- |
|  |  | Second assessment | | |
|  |  | Satisfied | Not very satisfied | Not satisfied at all |
| First assessment | Satisfied | 4340 ^a^ | 119 ^c^ | 3 ^c^ |
|  | Not very satisfied | 117 ^b^ | 122 ^d^ | 15 ^d^ |
|  | Not satisfied at all | 12 ^b^ | 15 ^d^ | 29 ^d^ |
| Values are presented as frequencies. | |  |  |  |
| a: This group was defined as "Consistently satisfied". | | |  |  |
| b: These groups were defined as "Improving". | | |  |  |
| c: These groups were defined as "Deteriorating". | | |  |  |
| d: These groups were defined as "Consistently unsatisifed". | | | |  |
